# Molecular Mechanisms of White Spot Syndrome Virus Infection and Perspectives on Treatments

**DOI:** 10.3390/v8010023

**Published:** 2016-01-18

**Authors:** Bas Verbruggen, Lisa K. Bickley, Ronny van Aerle, Kelly S. Bateman, Grant D. Stentiford, Eduarda M. Santos, Charles R. Tyler

**Affiliations:** 1 Biosciences, College of Life & Environmental Sciences, Geoffrey Pope Building, University of Exeter, Exeter, Devon EX4, UK; L.K.Bickley@exeter.ac.uk; 2 European Union Reference Laboratory for Crustacean Diseases, Centre for Environment, Fisheries and Aquaculture Science (Cefas), Weymouth Laboratory, Weymouth, Dorset DT4 8UB, UK; ronny.vanaerle@cefas.co.uk (R.V.A.); kelly.bateman@cefas.co.uk (K.S.B.); grant.stentiford@cefas.co.uk (G.D.S.)

**Keywords:** White Spot Syndrome Virus, host–pathogen interactions, viral infection pathway, endocytosis, stress responses, apoptosis, treatments, miRNA

## Abstract

Since its emergence in the 1990s, White Spot Disease (WSD) has had major economic and societal impact in the crustacean aquaculture sector. Over the years shrimp farming alone has experienced billion dollar losses through WSD. The disease is caused by the White Spot Syndrome Virus (WSSV), a large dsDNA virus and the only member of the Nimaviridae family. Susceptibility to WSSV in a wide range of crustacean hosts makes it a major risk factor in the translocation of live animals and in commodity products. Currently there are no effective treatments for this disease. Understanding the molecular basis of disease processes has contributed significantly to the treatment of many human and animal pathogens, and with a similar aim considerable efforts have been directed towards understanding host–pathogen molecular interactions for WSD. Work on the molecular mechanisms of pathogenesis in aquatic crustaceans has been restricted by a lack of sequenced and annotated genomes for host species. Nevertheless, some of the key host–pathogen interactions have been established: between viral envelope proteins and host cell receptors at initiation of infection, involvement of various immune system pathways in response to WSSV, and the roles of various host and virus miRNAs in mitigation or progression of disease. Despite these advances, many fundamental knowledge gaps remain; for example, the roles of the majority of WSSV proteins are still unknown. In this review we assess current knowledge of how WSSV infects and replicates in its host, and critique strategies for WSD treatment.

## 1. Introduction

Since its emergence in the early 1990s, White Spot Disease (WSD) has become the greatest threat to global crustacean aquaculture industries [1]. The first case of WSD was reported in China in 1991 [2] and this was followed by spread to other major aquaculture regions of the world including East and Southeast Asia, the Americas, India, the Middle East [2,3,4,5,6,7], and even Europe [8]. The total economic damage caused by WSD to the shrimp aquaculture industry has been estimated at $8–$15 billion since its emergence [9], increasing by $1 billion annually [10,11]. Annual losses have traditionally equated to approximately one tenth of global shrimp production [11]. A wide range of other crustacean hosts are susceptible to WSSV infection and disease. Natural populations of these hosts can also act as reservoirs for this pathogen [11,12]. Given the scale of WSD in captive crustaceans, it is not surprising that considerable scientific effort has been directed towards establishing the underlying mechanisms of the disease and identifying potential treatments for disease prevention or alleviation. However, despite a growing knowledge base and research interest, to date cost-effective vaccinations and/or treatments remain elusive.

WSD is caused by White spot syndrome virus (WSSV) [13], a double-stranded DNA virus and the only member of the genus *Whispovirus* and family *Nimaviridae* [14,15]. WSSV was originally classified as member of the *Baculoviridae* family, but it was later reclassified and named White Spot Syndrome Virus 1 by the International Committee on Taxonomy of Viruses ICTV [14,15,16,17]. The *Whispovirus* is a newly recognized family and its membership is likely to increase in the future as new taxa are discovered [17]. Vlak *et al.* [17] tentatively list B virus, B2 virus, τ (tau) virus, and Baculo-A and Baculo-B viruses as putative members of the *Whispovirus* genus and *Nimaviridae* family.

Animals suffering from WSD display various clinical signs including lethargy, reduced food consumption, reduced preening activities, a loosening of the cuticle, and a discoloration of the hepatopancreas [18,19]. White calcified spots appearing on the exoskeleton are diagnostic of WSD in some [19] but not all host species (e.g., the Indian prawn (*Penaeus indicus*) [20]). In farmed shrimp mortality is rapid and 3–10 days after infection cumulative mortality is generally between 90% and 100% [6,21].

**Figure 1:**
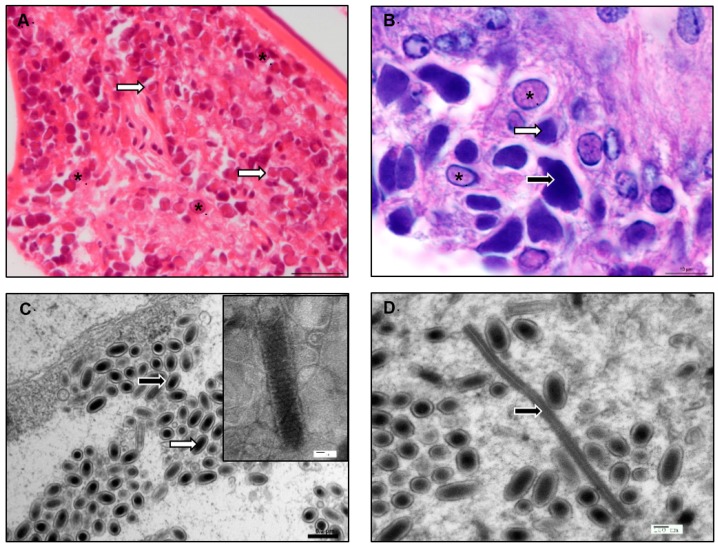
White Spot Syndrome Virus (WSSV) infection of *Litopenaeus vannamei*. The infection progresses through different stages that can be seen in the nucleus via histology. (**A**) Early-stage infected cells display enlarged nuclei with marginalized chromatin and a homogenous eosinophilic central region. These then develop an intranuclear eosinophilic Cowdry A-type inclusion (*); this can be surrounded by a clear halo beneath the nuclear membrane (**white arrow**). Scale bar = 25 µm; (**B**) The eosinophilic inclusion usually expands to fill the nucleus (*). This inclusion becomes basophilic in staining and denser in color as the infection progresses (**white arrow**). Nuclei then disintegrate so that the content fuses with the cytoplasm (**black arrow**). Scale bar = 10 µm. H & E stain; (**C**) WSSV virions appear ovoid in shape and contain an electron-dense nucleocapsid (**white arrow**) within a trilaminar envelope (**black arrow**). Scale bar = 0.2 µm. Inset. Negatively stained WSSV nucleocapsid, showing the presence of cross-hatched or striated material that is structured as a series of stacked rings of subunits and is a key diagnostic feature of WSSV. Scale bar = 20 nm; (**D**) Presumptive nucleocapsid material within the nucleus prior to envelopment. This material is cross-hatched or striated in appearance and linear prior to its incorporation in the formation of mature WSSV particles. This linear nucleocapsid material is observed sporadically in the manufacture of the WSSV particles. Scale bar = 100 nm. Transmission electron microscopy images.

WSSV infection occurs in all tissues of mesodermal and ectodermal origin (e.g., gills, lymphoid organ, cuticular epithelium, sub-cuticular connective tissues). Infected nuclei become hypertrophied with marginalized chromatin, and contain inclusion bodies that stain intensely eosinophillic in early-stage infection and basophilic in more advanced infection (Figure 1) [22]. Morphologically, WSSV virions are large and rod-shaped with dimensions in the range of 80–120 × 250–380 nm [13,23,24]. Structurally, they have a nucleocapsid surrounded by a trilaminar envelope [23] with a tail-like appendage, the function of which is unknown [13,23].

Economic drivers have focused research on WSD in farmed shrimp; however, the host range of WSSV includes many other decapod and non-decapod species including crabs, lobsters, prawns, crayfishes, and copepods [18,25]. To date 98 potential host species for WSD have been identified [2]. It is also the case that many organisms living in a WSSV-infected pond can function as a vector for WSSV [2]. Although WSD causes high levels of mortality in all cultured shrimps, it is not necessarily fatal to other hosts [26]. Variation in disease susceptibility occurs across the Crustacea and this is of particular interest as it may provide insights into disease resistance [3,27,28,29,30,31,32,33,34,35,36]. The shore crab (*Carcinus maenas*), for instance, while confirmed as susceptible to infection, appears to be especially recalcitrant to development of disease, showing little pathology and low mortality rates [36].

WSSV can be transmitted through the consumption of infected tissue, by cannibalism/predation, and via exposure to water containing WSSV virions [37,38]. Transmission through ingestion of contaminated prey tends to be the most effective path for infection of these two exposure routes [18,39]. A comparison between Asian tiger shrimp (*Penaeus monodon*) and Whiteleg shrimp (*Litopenaeus vannamei*) has shown that overall transmission rates are similar in these species but the relative contributions of direct and indirect transmission rates differ [40]. Transmission through physical contact with an infected animal is sufficient to initiate and progress WSD epidemics in ponds [40]. Vertical transmission (parents to progeny) of WSSV has been suggested and WSSV particles have been identified in oocytes. However, WSSV particles appear to be absent in mature eggs, suggesting that oocytes that contain the virus do not develop to mature eggs [18]. Vertical transmission might therefore occur through ingestion of viral particles that have been shed by adults during spawning into larval stages [18,37,41]. Virus pathogenicity can also be affected as it passes between species. As an example, WSSV, when passed through *Macrobrachium rosenbergii*, had a reduced pathogenicity (reduced mortality rates) in *P. monodon* when compared to non-transmitted virus. How this change in pathogenicity is mediated has not been established but was shown to be accompanied by variations in tandem repeat regions in the WSSV genome [42]. When considering transmission of WSSV between different geographical locations there is good evidence that this is facilitated by the transport of live and frozen uncooked shrimp [43,44] and the import of brood stock [11].

Prevention or treatment strategies for WSD disease could be advanced through an understanding of how this virus infects organisms and/or how relatively resistant animals process WSSV during the infection process. This requires understanding of the (molecular) interactions between WSSV and its potential hosts. In the infection process, WSSV invades host cells and initiates replication of its components. This is followed by assembly and release of new virions, resulting in host cell death and disease. To prevent disease, hosts must recognize the invading pathogen and elicit appropriate defense strategies or create a cellular environment that is not appropriate for production of new virions. A number of review articles have been published detailing the interactions between viruses and the host innate immune system (Li *et al.* [45], Shekhar and Ponniah [46], Sanchez-Paz [47], and Sritunyalucksana *et al.* [48]) but the interactions between WSSV and the host intracellular environment have received less attention. This is fundamental for advancing our understanding of the WSD infection process and exploring potential opportunities for disease treatment and prevention. In this review, we analyze the current knowledge on the WSSV genome, with a focus on the molecular mechanisms that enable WSSV to interact with host machinery and maintain a cellular environment favorable for the production of new virions. We then investigate the current treatment options that have been explored and consider possible future directions for advancing disease treatment and mitigation.

## 2. The WSSV Genome and miRNAS

### 2.1. WSSV Genome

WSSV contains a circular dsDNA genome of approximately 300 kb in size. Genome sequences for four WSSV isolates are available (a Chinese isolate (WSSV-CN; GenBank Accession AF332093) [49], an isolate from Thailand (WSSV-TH; GenBank Accession AF369029) [16], a Taiwanese isolate (WSSV-TW; GenBank Accession AF440570), and a Korean isolate (WSSV-KR; GenBank Accession JX515788) [50]). They differ in size, indicating some degree of genomic instability: (293 kb (Thailand), 296 kb (Korea), 305 kb (China), and 307 kb (Taiwan)). Overall, the sequence identity between isolates ranges between 97% and 99%, and the GC content in all isolates is 41% [50]. The WSSV genome contains nine homologous regions (*hr 1–9*) consisting of several repeats of 250–300 bp fragments, encompassing direct repeats, atypical inverted repeat sequences, and imperfect palindromic sequences [16]. Such *hr* regions in the genomes of baculoviruses have been hypothesized to play a part in DNA replication [51].

Several important genetic variations have been identified between the genomes of the Thai, Taiwanese, and Chinese isolates [51]. The largest involves a deletion of approximately 13 kb (WSSV-TH) and 1 kb (WSSV-CN), in the same genomic region, relative to WSSV-TW [51]. A region of approximately 750 bp shows variation between WSSV-TH and WSSV-TW, but the sequence of this region has no homology to sequences in publically available databases. Another variation includes a 1337 bp insert in WSSV-TH that shows 100% homology with a known transposable element [51]. Variable number tandem repeats (VNTR) are present amongst four *hr*s: *hr1*, *hr8*, *hr3* and *hr9*. VNTRs occur in open reading frames (ORF), e.g., *ORF75*, *ORF94* and *ORF125* in WSSV-TH, and have been suggested for use in genotyping WSSV [51,52]. Sequence variations have been shown to be stable over at least six passages through three different penaeid shrimp species [52]. Smaller variations like single nucleotide polymorphisms and short insertions/deletions are found throughout the WSSV genome [47]. The effects of genetic variation on WSSV virulence and fitness have been investigated in a number of studies, which have demonstrated higher virulence and competitive fitness in isolates with smaller genomes [53].

Putative ORFs in the WSSV genome have been determined for every isolate. These ORFs are present on both strands (~54% forward and ~46% reverse [16,50]), and the total number of estimated ORFs in the different isolates are 532 for WSSV-TW, 515 for WSSV-KR, 531 for WSSV-CN, and 684 for WSSV-TH. The nomenclature of ORFs differs in GenBank; for reference the conventions are as follows: WSSV-TW = WSSVxxx, WSSV-KR = wssv_xxxxx, WSSV-CN = wsvxxx and WSSV-TH = ORFxxx. The number of expressed ORFs is difficult to determine since many do not show homology with known protein sequences. Estimates are based on the potential to code for proteins of at least 50–60 amino acids. Functional predictions based on sequence similarity and protein motifs indicate that around 180 ORFs are likely to be expressed [16,49]. Microarrays constructed based on 184 putative ORFs of WSSV-TH confirmed expression of 79% of these ORFs in the gills of *P. monodon* infected with WSSV [54]. An analysis of the codons of ORFs (>100 codons) in WSSV-TW, WSSV-CN, and WSSV-TH indicated that codon usage bias and base composition are determined by compositional limitations and mutational pressure [55]. Interesting features of WSSV ORFs include one exceptionally long ORF—the longest among all viruses, specifically wssv_03600—which is 18,221 bp in the Korean isolate. Its protein product, VP664, is a major structural protein that forms the stacked rings of the WSSV nucleocapsid [16,50,56,57,58]. Furthermore, only around 30% of the ORFs in the WSSV genome contain a polyadenylation signal. ORFs without polyadenylation signals are usually part of a cluster of ORFs with small intergenic regions and identical transcriptional orientation that can produce polycistronic mRNAs (e.g., the *vp60b*/*wssv478*/*wssv479*/*vp28* cluster) [59,60,61,62]. Translation from these polycistronic mRNAs is likely to be facilitated by internal ribosome entry sites (IRES) [62,63]. Several IRES have been identified thus far including in the 5′ UTR of *wssv480* (*vp28*) [64], in the coding regions of *wssv396* (vp31) and *wssv395* (*vp39b*) [63], and in the 5′ UTR of *icp35* [62]. Translation initiation through IRES enables viral protein production even under unfavorable conditions such as during host response to viral infection, therefore making the virus more robust to host interference [63,65].

### 2.2. miRNAs, WSSV Infection, and Pathogenesis

MiRNAs (small non-coding RNA molecules) have been documented to be widely expressed in both animal and plant species, and have key regulatory roles in a number of cellular pathways, including early development, cell differentiation and proliferation, apoptosis, signal transduction, and immunity [66].

It is now well established that miRNAs are involved in many host–pathogen interactions, as reviewed in Asgari 2011 and Skalsky *et al.* 2010 [67,68]. The primary function of miRNAs involves regulation of gene expression at the post-transcriptional level. Typically, miRNAs bind to complementary sequences (either partial or complete) in the mRNA of target genes, regulating gene expression by repressing translation or directing sequence-specific degradation of the mRNA. However, evidence has also emerged that interaction of miRNAs with target genes may also function to induce gene expression [69,70,71]. MiRNAs encoded in a virus genome may function to regulate either viral or host genes to manipulate immune responses and cellular functions for the benefit of the virus. Additionally, viruses may regulate and use host miRNAs to facilitate their own replication or to regulate the virus life cycle [72,73]. Alternatively, host miRNAs can act to limit viral replication or alter cellular processes to the disadvantage of the virus [68,74].

In WSSV-challenged shrimp (*M. japonicus*) 63 host miRNAs have been identified through small RNA sequencing, of which 48 could be mapped to other known arthropod miRNAs in the miRBase database [75]. Thirty-one of the miRNAs show differential expression in response to WSSV infection. Using target gene prediction algorithms, many of the miRNAs were predicted to target genes involved in host immunity, including the small GTPase-mediated signaling transduction pathway, autophagy, phagocytosis, apoptosis, the Toll-like receptor signal pathway, antimicrobial humoral response, endocytosis, RNAi, and regulation of the innate immune response. Huang and Zhang [76] showed a more direct interaction between host miRNA and WSSV: a miRNA found to be upregulated in WSSV-infected *M. japonicus*, miR-7, was predicted to target the 3′-untranslated region of *wsv477*. In insect High Five cells, the expression of enhanced green fluorescent protein (EGFP) was dramatically reduced when coupled to the *wsv477* 3′UTR compared to controls [76]. *Wsv477* is an early gene that is involved in viral gene replication. Its inhibition would thus have negative effects on WSSV replication. Indeed, injection of miR-7 was shown to reduce WSSV copies 1000-fold compared with WSSV only at 72 and 96 h post-infection [76].

The specific viral miRNAs encoded by WSSV have been investigated in *M. japonicus* [77,78]. In the first study on these miRNAs in *M.* *japonicus*, WSSV was shown to have the capacity to encode 40 distinct miRNAs, which is a miRNA density 360 times greater than in humans [77]. The authors of that work suggested that this high miRNA content may contribute to the ability of viruses to respond rapidly to selective pressures placed on them from the host organism. In this study miRNAs were first predicted through bioinformatic analyses and subsequently their presence was confirmed through expression (microarray) studies and Northern blots. Subsequent work applying small RNA sequencing identified additional WSSV miRNAs [78], bringing the total number of WSSV miRNAs collectively to 89 [78]. It was observed that the majority of the miRNAs were expressed during the early stages of infection and that host genes like *Drosha*, *Dicer1*, and *Ago1* are necessary for successful miRNA biogenesis [77,78]. Interestingly, several miRNAs showed differential expression across tissue types, indicating that viral regulatory strategies could be regulated to fit the infected tissue type [78]. One example of this regulatory effect was the potential of WSSV-miR-N24 to inhibit apoptosis through downregulation of caspase 8 expression [78]. WSSV miRNAs can also regulate the balance between promotion of viral infection and latency. He *et al.* [79] identified two WSSV miRNAs, WSSV-miR-66 and WSSV-miR-68, that promote WSSV infection through regulating expression of WSSV genes. It is hypothesized that the targets of these miRNAs, *wsv094* and *wsv177* (WSSV-miR-66), and *wsv248* and *wsv309* (WSSV-miR-68), are related to latency. These studies provide key evidence that WSSV miRNAs function as regulatory factors involved in the virus life cycle as well as the host immune response.

## 3. WSSV Infection

**Figure 2:**
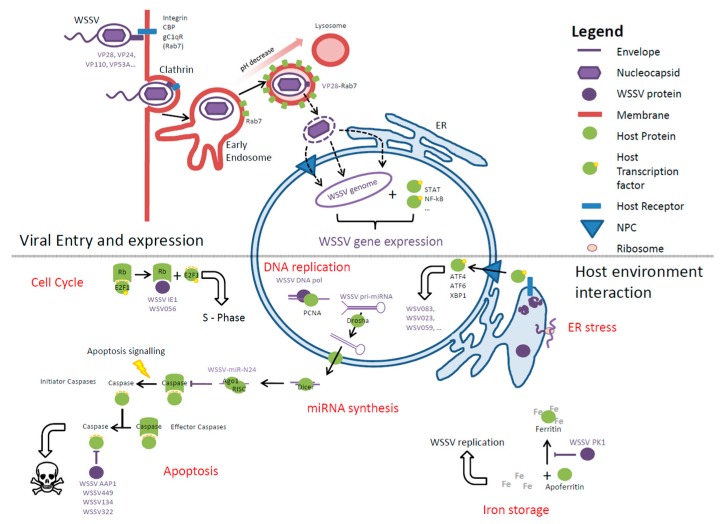
Overview of WSSV entry and environment interactions. Top panel: Viral entry into the host cell. WSSV proteins interact with host receptors, which leads to induction of Clathrin-mediated endocytosis. WSSV then travels through endosomes. During maturation the pH decreases, a cue for viruses to exit the endosomes. This stage probably involves an interaction between VP28 and Rab7. How WSSV passes through the nuclear envelope is unknown. Once in the nucleus, host transcription factors bind the WSSV genome (e.g., *ie1*) and initiate expression of viral genes. Bottom panel: Intracellular interactions between WSSV and the host cell. WSSV DNA replication requires host machinery (e.g., processivity factors) and to make these available WSSV can act to halt the cell cycle in the S-phase through E2F1. A high level of viral protein production can lead to ER stress, e.g., activation of unfolded protein response (UPR) pathways. Transcription factors of the UPR can activate expression of viral genes, which in turn may inhibit translation through eIF2. WSSV replication requires essential nutrients including iron. To prevent the host from withholding iron, WSSV can inhibit the binding of iron to Ferritin. WSSV can influence apoptosis signaling either through miRNA-mediated inhibition of initiator caspases or through viral proteins that inhibit effector caspase activity.

The life cycle of viruses is well studied, and can be broadly divided into three phases: entry into the host cell (either directly or through host mechanisms such as endocytosis), uncoating of the genome followed by replication, and, finally, particle assembly and release. A model for the WSSV life cycle and morphogenesis was suggested by Escobedo-Bonilla and colleagues [80]. Throughout these phases a wide range of molecular interactions occur between the WSSV and its host (see Figure 2). These molecular interactions can be key factors in determining host susceptibility and pathogenicity, and also provide opportunities for treatment interventions.

### 3.1. Viral Receptors, Interactions, and Entry

The primary obstacle for entry into host cells is the host’s cell membrane. Viruses have evolved several methods to overcome this barrier including via lipid fusion and membrane perforation, but they enter the host cell predominantly by endocytosis [81]. In the latter case viruses bind with host cell surface proteins, carbohydrates, and lipids [81], which then trigger one of the various endocytic pathways including Clathrin-mediated endocytosis, caveolar endocytosis, and macropinocytosis [81,82,83,84]. WSSV enters the host cell through activating the endocytotic process with protein interactions. Known interacting elements for WSSV and its hosts are presented in Table 1. It can be seen that VP28, the major envelope protein, is a major player in host–virus protein interactions [85,86,87,88]. Some interactions are beneficial for the virus; others have negative impacts. There is also a series of interacting proteins for which the functions are not well established.

**Table 1:** Established WSSV–host protein interactions.

Viral Protein	Host Protein	Species	Reference
VP24, VP32, VP39B, VP41A, VP51B, VP53A, VP53B, VP60A, VP110, VP124, VP337	Chitin-binding protein (PmCBP)	*Penaeus monodon*	[89,90]
VP53A	Glut1	*P. monodon*	[91]
VP15, VP26, VP28	gC1qR (PlgC1qR)	*Pacifastacus leniusculus*	[92]
VP95, VP28, VP26, VP24, VP19, VP14	C-type lectin (LvCTL1)	*Litopenaeus vannamei*	[93]
VP28	C-type lectin (FcLec3)	*Fenneropenaeus chinensis*	[94]
VP26, VP28	C-type lectins (MjLecA, MjLecB, MjLecC)	*Marsupenaeus japonicus*	[95]
VP28	C-type lectins (MjsvCL)	*M. japonicus*	[96]
VP28	C-type lectins (LdlrLec1, LdlrLec2)	*M. japonicus*	[97]
VP187	β-Integrin	*P. japonicus*	[98]
		*P. clarkii*	
VP26, VP31, VP37, VP90, VP136	β-Integrin	*L. vannamei*	[99]
WSSV-CLP	α-integrin, β-integrin, Syndecan	*F. chinensis*	[100] (Bioinformatic prediction)
VP15, VP28	Calreticulin (PlCRT)	*P. leniusculus*	[101]
VP466	Rab (PjRab)	*P. japonicus*	[102]
VP28	Rab7 (PmRab7)	*P. monodon*	[103]
ORF514	PCNA (lvPCNA)	*L. vannamei*	[104] (Bioinformatic prediction)
WSSV PK1	Ferritin (lvFerritin)	*L. vannamei*	[105]
Wsv083	FAK (MjFAK)	*M. japonicus*	[106]
AAP1 (WSSV449)	Caspase (PmCaspase)	*P. monodon*	[107]
WSSV134, WSSV332	Caspase (PmCasp)	*P. monodon*	[108]
WSSV249	Ubc (PvUbc)	*L. vannamei*	[109]
ICP11	Histones	*P. monodon*	[110]
VP9	RACK1 (PmRACK1)	*P. monodon*	[111]
VP15	FKBP46 (PmFKBP46)	*P. monodon*	[112]
VP15	CRT (PlgCRT)	*P. leniusculus*	[101]
WSSV-miRNA	Dorsha, Dicer, Ago1	*–*	[77]
VP14	Arginine kinase (LvAK)	*L. vannamei*	[113]
VP26	Actin	*Procambarus clarkii*	[114]
ORF427	PPs	*L. vannamei*	[115]
WSSV IE1	TATA box-binding protein (PmTBP)	*P. monodon*	[116]
WSSV IE1, WSV056	Retinoblastoma protein (Lv-RBL)	*L. vannamei*	[117]

Integrin receptors on host cell surfaces have been shown to be important targets for WSSV. Principally involved in the binding of cells to the extracellular matrix (or cell–cell adhesion), integrins are heterodimeric surface receptors that recognize Arg-Gly-Asp (RGD) motifs in target proteins [118]. Several WSSV proteins can bind to α- or β-integrin homologues. Immunoprecipitation experiments have shown binding of β-integrin by VP187 (wsv209), a viral protein that contains an RGD motif [98]. This body of work also found that WSSV infection could be blocked by soluble integrin, integrin-specific antibody, an RGD-containing peptide, and silencing of β-integrin, indicating an important role for integrins as WSSV receptors [98]. The viral proteins VP26, VP31, VP37, VP90 and VP136 also interact with the integrin receptors of *L. vannamei*, mainly through interactions with RGD-, YGL-, and LDV peptide motifs present in these viral proteins [99]. VP26 is a tegument protein that associates with a protein complex involving VP24, VP28, VP38A, VP51A, and WSV010 in the viral envelope [57,119]. Since VP26 is not present on the cell exterior it is unlikely to play a role in binding host cells. Finally, bioinformatics analyses have predicted that WSSV collagen-like protein (WSSV-CLP) interacts with integrins on the basis of sequence similarity to known interacting protein pairs [100]. Together, these studies show that multiple proteins are involved in the recognition and binding of host integrins [99]. In *P. monodon*, the cell surface Chitin-binding protein (PmCBP) has been shown to bind to 11 WSSV proteins that likely form a complex on the surface of WSSV (VP24, VP110, VP53A, VP53B, VP337, VP32, VP124, VP41A, VP51B, VP60A, and VP39B) [90]. Facilitating viral entry through protein complex interactions also occurs for other enveloped DNA viruses (e.g., Herpesviridae [120]). Interactions of the viral protein complex with PmCBP and with VP53A appear to be facilitated by glucose transporter1 (Glut1) [91].

The innate immune system of the host employs groups of proteins that are able to recognize and bind pathogen-associated molecular patterns (PAMP) molecules. One family of proteins that recognizes non-self molecules are the lectins. Calcium-dependent lectins (C-type lectins) have been demonstrated to interact with WSSV proteins. Several interactions between host C-type lectins and WSSV proteins have been identified (see Table 1), including a C-type lectin in *L. vannamei* (LvCTL1) that can interact with VP95, VP28, VP26, VP24, VP19, and VP14 [93]. Treating WSSV with recombinant LvCTL1 prior to a shrimp WSSV exposure was shown to result in higher survival rates, indicating a protective effect [93]. Lectins from other shrimp species have shown interactions with VP26 and VP28. For example, in the Chinese white shrimp (*F. chinensis*) a lectin (FcLec3) has been identified that can interact with VP28 [94]. Using VP26, VP28, and VP281 to screen a phage display library of *M. japonicus*, three lectins (MjLecA, MjLecB, and MjLecC) were identified to interact with the viral proteins [95]. Of these, MjLecA and MjLecB were shown to reduce viral infection rate *in vitro* [95]. Interactions with other lectins found in *M. japonicus* indicate a contradictory relationship between WSSV and lectins. For example, a C-type lectin isolated from the stomach of *M. japonicus* (MjsvCL) has been shown to interact with VP28 [96] but, in contrast to other lectins, MjsvCL appears to facilitate WSSV infection. MjsvCL expression is induced by viral infection, and inhibition of its expression by RNAi results in lower virus replication, whereas exogenous MjsvCL enhances replication [96]. MjsvCL contributes to viral entry by binding of VP28 and the calreticulin receptor on the cell surface. MjsvCL thus works as a bridge between the virus and the calreticulin receptor [96]. The dual roles of lectins are indicative of the arms race between the virus and the host immune system, as is also observed for various viral pathogens in humans [121]. Members of the complement system have also been shown to interact with WSSV proteins. In the signal crayfish *Pacifastacus leniusculus* the surface receptor for C1q (a component of the complement system) can interact with WSSV proteins VP15, VP26, and VP28 [92] and this is upregulated upon WSSV infection, producing a protective effect.

After binding to receptors on the cell surface, the enveloped virus can either penetrate the membrane directly or undergo uptake through endocytosis. Since there are several routes of endocytosis it is useful to identify which of these may be adopted by WSSV, if any. Several experiments have investigated WSSV endocytosis and the results depend, at least in part, on cell type. Huang *et al.* [101] showed that WSSV can enter hemocytes of *L. vannamei* in primary culture through caveolar endocytosis. The evidence for this included inhibition of WSSV uptake under methyl-β-cyclodextrin (MβCD, an inhibitor of caveolar endocytosis) treatment and a lack of effect of chlorpromazine (CPZ, an inhibitor of Clathrin-mediated endocytosis) [122]. There were similar findings in the crayfish, *Cherax quadricarinatus* [123]. However, in crayfish hematopoietic tissue (HPT) it appears to be the Clathrin-mediated endocytosis pathway that is responsible for viral uptake [124]. For this tissue, CPZ significantly inhibited WSSV internalization. Furthermore, WSSV particles co-localized with Clathrin and there was a dependence on membrane cholesterol and dynamin supported uptake, indicating Clathrin-mediated endocytosis. It is difficult to reconcile this apparent contradiction in uptake pathways; however, Huang *et al.* [124] point to data suggesting that MβCD also affects Clathrin-mediated endocytosis and further suggesting that WSSV could employ more than one endocytosis pathway for host entry.

### 3.2. Escaping from Endosomes

After penetrating the cellular membrane, WSSV particles become localized within early endosomes. Cellular cargo carried by endocytic vesicles can have a variety of different destinations in the cell depending on the sorting that occurs in the early endosome [125]. Some proteins or lipids are recycled to the plasma membranes, while others are degraded in lysosomes. Viruses need to avoid this fate. The sorting process occurs within minutes of vesicular entry into the cell. Due to their size, viruses are sorted by the host to the degradation pathway [126]. In completing their primary roles in sorting the endocytotic cargo, early endosomes “mature” in a process that includes several changes to the organelles including lumen acidification, their movement toward the perinuclear region, and changes in membrane lipid/protein composition. It is this maturation process that provides the cue for viruses to initiate their escape from the endosomes, which involves conformational changes of viral proteins in response to a lower pH. Escape from the endosomes can occur either through membrane fusion or lysis/leakage of the endosomal compartments [127]. After release from the endosomes the virus directs itself to the nucleus and penetrates the nuclear envelope. The pathways adopted to accomplish this by various virus families are reviewed by Kobiler *et al.* [128], the main difference being whether the nuclear pore complex (NPC) is involved or not.

An important class of regulatory proteins in endocytosis are the small Rab GTPases. Rab GTPases are key regulators of endosome maturation. Early endosomes are characterized by the presence of Rab5 on their membranes [129]. During the endosome maturation process Rab5 is exchanged for Rab7, the Rab GTPase associated with late endosomes, and this operates in a positive feedback loop mechanism [130]. Rab7 can subsequently associate with RAB effector proteins like RILP that bind Dynein, ensuring movement of the vesicle to the perinuclear area [129].

The Rab GTPases have been implicated in the WSSV infection process. In *P. monodon* Rab7 (PmRab7) has been shown to interact with WSSV protein VP28 [103]. Moreover it has been shown that in shrimp injected with WSSV and PmRab7, or PmRab7 antibody, there is a decreased rate in mortality to 15% and 5%, respectively, compared with a mortality rate of 95% in animals injected with WSSV alone [103]. In *L. vannamei* brood stock, dsRNA-mediated silencing of *lvRab7* has also been shown to reduce mortality rates, albeit a mild reduction [131]. Similarly, in *P. monodon* silencing of both viral ribonucleotide reductase small subunit (*rr2*) and host *Rab7* resulted in a 95% survival of infected animals compared with 100% mortality in animals treated with WSSV only [132,133]. It is unclear how the interaction between VP28 and Rab7 is mediated as the proteins are separated by the endosomal membrane. It is possible that this interaction occurs after WSSV has been released from the endosomes, but this can occur only if its envelope containing VP28 remains attached to the nucleocapsid. Alternatively, the Rab proteins could be present on the cell surface [103]. Applying subtractive hybridization, the amplification of only differentially expressed genes has shown that a Rab GTPase gene was upregulated in the hepatopancreas of WSSV-resistant *P. japonicus* [134]. This Rab GTPase gene showed homology with Rab6A, a protein involved in transport between the endosomes, Golgi, and endoplasmic reticulum [135]. It is possible that a higher expression of Rab6A leads to an increase in recycling of endosomes, removing the potential for WSSV to interact with Rab7, which in turn inhibits infection and thereby leads to resistance.

Overall, the mechanisms through which WSSV reaches the nucleus after its initial uptake by caveolar-mediated endocytosis remain poorly understood. Since the journey from the endosomes to nucleus is a limiting step in the infection process for many viruses, a greater knowledge of this pathway for WSSV is likely to help identify targets for drug development for disease prevention [128].

### 3.3. Viral Replication—The Molecular Processes

Once inside the host nucleus the virus has to express its own genes to allow for its own replication. Since WSSV does not carry its own transcriptional machinery, it relies initially on the host to supply these. Host transcription factors can bind to viral promoters and activate transcription. Genes expressed this way are typically named immediate early genes. These genes encode transcription factors and other regulators that enable transcription of viral genes. Genes dependent on the expression of the immediate early genes are classified as “early genes”. So-called “late genes” are expressed after initiation of viral DNA synthesis and typically include structural proteins [47]. A list of known interactions between host proteins and viral genes is provided in Table 2.

**Table 2:** Established WSSV–host gene expression interactions.

Transcription Factor (Host, Virus)	Target (Host, Virus)	Species	Reference
STAT (PmSTAT)	*ie1*	*Penaeus monodon*	[136]
PHB2 (Sf-PHB2)	*ie1*	*Spodoptera frugiperda*	[137]
Nf-κB (LvRelish, LvDorsal)	*ie1*, *WSSV303*, *WSSV371*	*Litopenaeus Vannamei*	[138,139,140]
c-JUN	*ie1*	*L. vannamei*	[141] (Bioinformatic prediction)
XBP1 (LvXBP1)	*wsv083*	*L. vannamei*	[142]
ATF4 (LvATF4)	*wsv023*	*L. vannamei*	[142]
KLF (PmKLF)	*WSSV108*	*P. monodon*	[143]
ATFβ (LvATFβ)	*wsv059*, *wsv166*	*L. vannamei*	[144]
VP38, VP41B	Caspase (PjCaspase)	*M. japonicus*	[145]
WSSV-miR-N24	Caspase 8	*M. japonicus*	[78]

Viral protein/gene/miRNA is underlined.

Immediate early genes expressed upon WSSV infection have been identified in infected crayfish hemocytes via the use of a protein synthesis inhibitor, cycloheximide (CHX) [146]. Inhibiting protein synthesis disables the ability of WSSV to create its own transcription factors and thus relies solely on host factors. Using this approach a total of 16 ORFs have been identified including WSV069 (IE1), *WSV051*, *WSV100*, *WSV079* with transactivation activity, *WSV083* with Ser/Thr kinase domain, and *WSV249*, believed to function as an ubiquitin E3 ligase [146]. In the Taiwan isolate of WSSV three WSSV ORFs (*WSSV126*, *WSSV242*, and *WSSV418*) have been identified that were insensitive to CHX treatment and were subsequently named *immediate early 1* (*ie1*), *ie2*, and *ie3*, respectively [147]. Further experiments established that the promoter of *ie1* could express EGFP in the fall army worm, *Spodoptera frugiperda*, Sf9 cells indicating that *ie1* can be activated even by non-decapod host transcription factors. Other work has shown that the *ie1* promoter of WSSV is one of the most inducible promoters in insect cells [148] and can regulate the expression of genes in mammalian cells also [149]. It has been suggested that the broad expression capabilities of *ie1* are a reason for the wide host range of WSSV [147]. Characterization of this promoter has revealed that *ie1* contains an initiator element, TATA-box, and a binding site for the transcription factor Sp1 [137]. Activation studies of the *ie1* promoter in natural hosts of WSSV have shown that it can be activated by various host factors.

In *P. monodon*, STAT (PmSTAT) can increase the activity of the *ie1* promoter through a STAT-binding motif [136]. Interestingly, STAT is part of the JAK-STAT anti-viral signaling pathway, indicating that WSSV can hijack the host immune response in order to promote expression of its own genes. Knockdown experiments of a cytokine receptor that activates the JAK-STAT pathway in *L. vannamei* (*LvDOME*) resulted in lower cumulative mortality and fewer WSSV copies, providing further evidence for the interaction between JAK-STAT and WSSV [150].

In addition to STAT, WSSV can hijack other immune-related pathways, notably Nuclear Factor-κ-B (NF-κB) signaling and MAP kinase signaling. NF-κB is a key regulator of the immune response and important for cell survival [151]. The *ie1* promoter contains a binding site for the NF-κB family of proteins [139]. In *L. vannamei* a homologue of NF-κB, LvRelish, can bind to the putative NF-κB binding site in the *ie1* promoter [139]. Other *in vivo* experiments confirm that NF-κB homologues lvDorsal and LvRelish can stimulate the expression of WSSV genes (e.g., *WSSV069*, *WSSV303*, and *WSSV371*) through interaction with *ie1* [140]. Furthermore, the expression of these transcription factors is upregulated upon WSSV infection [138,140]. The WSSV genome also encodes a protein (WSSV449) that shows similar functionality to Tube, a component of the NF-κB pathway. WSSV449 activates the host NF-κB pathway and through that system promotes expression of viral genes. WSSV, as for many other viruses (e.g., HIV-1 and hepatitis B), thus employs the NF-κB pathway for its own benefit [138]. The MAP kinase c-Jun N-terminal kinase (JNK) also has an ability to bind the *ie1* promoter, implicating involvement of MAP kinase signaling in WSSV gene expression. In *L. vannamei*, silencing of *LvJNK* with dsRNA resulted in decreased viral proliferation, and specific MAP kinase inhibitors delay viral gene expression [141]. A potential binding site for c-JUN has been identified in the *ie1* promoter via sequence similarity to c-JUN binding sites in TRANSFAC, a database of eukaryotic transcription factors and their binding sites. However, transcription factor binding has yet to be validated experimentally [141,152]. After expression of *ie1*, WSSV IE1 protein (a viral transcription factor) is then able to facilitate expression of the viral early genes. WSSV IE1 has been shown to accomplish this through cooperation with *P. monodon* TATA box-binding proteins (PmTBP) in transcription initiation [116].

*WSSV108* (a probable transcription factor/activator and/or regulator through SUMOylation) is another WSSV immediate early gene [153]. Liu *et al.* [143] used the transcription factor binding site databases TRANSFAC and JASPAR to identify regulatory elements upstream of *WSSV108*, and found elements for Sp1/KLF, GATA-1, C/EBP, c-Myc, and AP-1. Furthermore, it was confirmed that recombinant Krüppel-like factor from *P. monodon* (rPmKLF) could bind to the KLF element and that a deletion in this element had the largest impact on expression through the *WSSV108* promoter [143]. The action of these transcription factors result in a higher expression level of the *WSSV108* promoter than *ie1* [143,146,153].

Expression of genes in the different phases of the infection process for WSSV has been reviewed previously by Sánchez-Paz [47]. Briefly, the genes can be grouped according to their function and they comprise of at least three classes: transcription factors, kinases, and ubiquitin E3 ligases [47]. Early phase genes target replication of the viral genome and include DNA polymerases, DNA helicases, and genes involved in nucleic acid metabolism [47]. After genome replication the functional characteristics of expressed genes shifts to structural proteins and to particle assembly during the late phase [47].

Different viruses exploit different phases of the host cell cycle for viral genome replication. Some arrest cells in G0, G1, or at the G1/S boundary so host DNA replication is prevented, whereas others tend to favor the S-phase where host DNA replication machinery is widely available [117]. Those that arrest host cells in G0/G1/G1/S, which include the herpes simplex virus and Epstein–Barr virus, often carry DNA replication machinery in their genomes instead of relying on the host [117,154,155]. WSSV appears to operate in the S-phase and depend on host factors for replication. Viruses often control the host cell cycle through interactions with proteins of the retinoblastoma (Rb) family, which are central regulators of the cell cycle, operating through interaction with E2F transcription factors [117]. WSSV IE1 and WSV056 (two paralogue genes) have been shown to be able to bind to an Rb-homologue in *L. vannamei* (lv-RBL), thereby possibly activating E2F1 and leading to S-phase entry. Indeed, overexpression of these two viral proteins in *Drosophila* S2 cells resulted in an increased portion of S-phase cells and a correlated decrease in G0/G1-phase cells [117]. The authors deduced that although WSSV carries some DNA replication machinery, it still relies on host factors; they therefore propose that WSSV utilizes the DNA replication machinery present in host cells during S-phase and promotes S-phase arrest to support successful replication of the viral genome.

Examples of host factors required for efficient replication are processivity factors. Processivity is the ability to catalyze consecutive reactions without releasing the substrate. While the DNA polymerase encoded in the WSSV genome (*WSV514*, [156]) shows polymerase activity [157], the average number of nucleotides added per DNA association event has been shown to be low [157]. Usually, processivity of DNA polymerases is improved by association with processivity factors, e.g., DNA clamps. WSSV does not encode its own processivity factors, which indicates that WSSV DNA polymerase might employ host processivity factors. Interaction of DNA polymerases with processivity factors occurs through a PIP-box motif. This motif is present on WSSV DNA polymerase [157]. To investigate the possibility of interaction with a host processivity factor in *L. vannamei*, the 3D structure of Proliferating Cell Nuclear Antigen (LvPCNA) was elucidated [104,158]. Likely PIP-box interaction models between LvPCNA and WSSV DNA polymerase were established but have yet to be confirmed experimentally.

### 3.4. Maintaining the Host Cell Environment

The presence of a virus within a host cell places demands on that cell, e.g., through draws on energy for anabolic reactions, demand for essential nutrients, and accumulation of non-host proteins. Such impacts on the cell lead to a deterioration of the cellular environment, making it less conducive for viral replication. Host cells also have evolved mechanisms to reduce the ability of viruses to replicate, for example through withholding nutrients in cases of infection or inhibiting translation. The cell might enter apoptosis, preventing further viral replication by undergoing self-destruction. To counteract these adverse cellular responses, viruses have evolved ways to interact with host metabolism, stress response systems, and apoptosis signaling for the purpose of retaining an appropriate environment for replication. Some of these interactions have been investigated for WSSV infections, but the limited understanding of the function of many WSSV proteins limits interpretation for some of the effects identified (Figure 2).

#### 3.4.1. Metabolism

The replication of the viral genome and synthesis of its structural components require a large amount of energy. To supply the cell with such large quantities of energy, host metabolism can be directed to induce aerobic glycolysis [159]. Often observed in cells during rapid proliferation, a high rate of glycolysis provides both energy and glycolytic intermediates that can be used in a variety of anabolic reactions [159]. This metabolic shift was originally described in cancer cells and is known as the Warburg effect [160]. The Warburg effect also encompasses enhancement of the pentose phosphate pathway, amino acid metabolism, and lipid homeostasis [161]. Induction of a Warburg-like effect by WSSV infection has been observed in *L. vannamei* hemocytes [162]. In a study by Su *et al.* [139], it was established that the PI3K-Akt-mTOR pathway was the mechanism through which metabolic changes where induced by WSSV. PI3K-Akt-mTOR is also employed by cancer cells and human papillomavirus to induce the Warburg effect [161,163,164]. However, the viral factors that interact with the host PI3K-Akt-mTOR pathway have yet to be identified.

#### 3.4.2. Iron

In addition to energy, WSSV and other invading pathogens also require essential nutrients including iron. In response to this, hosts have evolved mechanisms through which they can withhold iron from pathogens, thereby inhibiting their proliferation. The protein responsible for this mechanism is ferritin [105]. The ferritin defense mechanism has been demonstrated in *L. vannamei*, where its injection reduced susceptibility to WSSV infection. Higher amounts of ferritin resulted in a greater binding of iron and thus less iron available for WSSV proliferation. Conversely, dsRNA-mediated knockdown of ferritin resulted in a three-fold increase in viral copy number [165]. Through the use of a yeast two-hybrid experiment, it has been shown that WSSV protein kinase 1 (WSSV PK1) can interact with host ferritin and influence the availability of iron [105]. Binding of WSSV PK1 does not release iron from ferritin but rather prevents iron from binding to apoferritin (ferritin without bound iron). Injection of dsRNA specific to *WSSV PK1* decreases cumulative mortality, showing that disrupting host iron withholding mechanisms is important to successful WSSV infection [166].

#### 3.4.3. Endoplasmic Reticulum Stress Responses

The presence of a virus within the cell can induce the unfolded protein response (UPR), a cellular stress response activated because of accumulation of misfolded protein at the ER. This leads to phosphorylation of transmembrane kinases and activation of transcription factors. Outputs of the UPR include global translation shutdown, arrest of cell cycle, increase in expression of chaperone genes to aid in protein folding, and potentially apoptosis [167]. While increased folding capabilities can aid the virus, translation attenuation and apoptosis have adverse effects on viral replication [168]. Therefore many viruses have evolved mechanisms through which the host UPR response can be manipulated [169].

There are three pathways that can initiate the UPR response, namely via Inositol-requiring enzyme-1 to X-box binding protein 1 (IRE1-XBP1), via double-stranded RNA-activated Protein kinase-like ER kinase and Activating Transcription Factor 4 (PERK-ATF4), and via Activating Transcription Factor 6 (ATF6). Interactions between viruses and these UPR pathways are complex, as some viruses inhibit responses whereas others induce them. For example, the Epstein–Barr virus (EBV) induces UPR to aid in lytic replication [170], Rotavirus induces and controls UPR to prevent ER stress-related cell death [171], and Herpes simplex virus-1 inhibits UPR to maintain an environment that permits expression of viral genes [172,173].

Interactions between WSSV and UPR in shrimp have been demonstrated through studies on the expression of chaperone proteins [174,175,176,177,178]. Induction of the IRE1-XBP1 UPR pathway upon WSSV infection in *L. vannamei* has been shown through enhanced expression of *LvXBP1.* Furthermore, WSSV appears to benefit from its induction, as demonstrated by lower cumulative mortality following dsRNA-mediated knockdown of *LvXBP1* [173]. A potential mechanism for this effect is LvXBP1-mediated upregulation of expression of the viral gene *wsv083*, a predicted protein kinase 2, and *wsv023*, of unknown function [49,142]. Wsv083 has been shown to inhibit focal adhesion kinase, a regulator of innate immune system signaling [106,142]. The transcription factors of the PREK-ATF4 pathway can also induce expression of WSSV genes, depending on the presence of an ATF/CRE in the promoter (15 in total: *wsv023*, *wsv049*, *wsv064*, *wsv069*, *wsv138*, *wsv242*, *wsv256*, *wsv282*, *wsv303*, *wsv306*, *wsv313*, *wsv321*, *wsv343*, *wsv406*, and *wsv453*). In *L. vannamei* the UPR transcription factor LvATF4 (as for LvXBP1) also upregulates expression of *wsv023* [142].

The shutdown of translation following UPR activation is achieved through phosphorylation of the translation initiation factor subunit eIF2α [179]. Phosphorylated eIF2α inhibits activation of eIF2 by preventing the exchange of GDP for GTP by its eIF2β subunit. During WSSV infection in *L. vannamei* the level of LveIF2α decreases and the phosphorylation ratio increases, suggesting that the virus indeed initiates eIF2α-mediated translation inhibition [180]. Furthermore, adding an inhibitor of eIF2α phosphatases decreases viral loads, indicating that WSSV requires active eIF2 for successful replication. Xu *et al.* [180] suggest that WSSV could code for proteins able to prevent phosphorylation of eIF2α, thereby halting UPR initiated translation inhibition, a strategy that is also observed in the Chikungunya virus. In summary, data to date suggest that the presence of WSSV induces the UPR and is capable of interacting with downstream effectors and transcription factors activated through the UPR. These processes aid in the transcription of viral genes. However, limited knowledge of viral protein function limits our current understanding on the roles of these viral genes.

#### 3.4.4. Apoptosis

Cellular responses to stress can result in the induction of apoptosis of the host cell. Through apoptosis organisms can remove cells that are potentially harmful to their own health. Apoptosis is an important defense mechanism against abnormalities in cell programming and disease infection, including viruses. The relationship between virus and apoptosis is complex and viruses can influence the host apoptosis system by either inhibiting or inducing it. Inhibition is necessary to keep a cellular environment conducive to the production of new virions [181]. In later stages of viral infection, however, some viruses might induce apoptosis as a means of leaving cells and spreading further throughout the host [182]. Apoptosis has been documented to occur in *L. vannamei*, *P. monodon*, and *M. japonicus* cells infected with WSSV [183,184,185,186]. Leu *et al.* [187] proposed a detailed model for the apoptotic interaction between WSSV and shrimp, in which invasion of WSSV leads to activation of signaling pathways that increase expression of pro-apoptosis proteins (e.g., Caspases and voltage-dependent anion channels), membrane permeabilization of mitochondria, and increased oxidative stress. These molecular pathways lead to the initiation of the apoptosis program. In parallel, WSSV anti-apoptotic proteins attempt to block apoptosis and thereby keep the cell viable for replication. The balance between the pro- and anti-apoptosis activation processes will determine the fate of the WSSV-infected cell [187].

Caspases are key regulators of apoptosis and therefore important targets for viruses. The family can be generally grouped into initiator caspases and effector caspases. The first are activated through autocatalytic processes, whereas the latter are present as zymogens activated through cleavage by the initiator caspases [188]. Activation of effector caspases leads to an accelerated feedback loop of effector caspase activation and eventually permits the controlled destruction of cellular components [189]. WSSV proteins show interactions with shrimp caspases. Direct inhibition of the activity of a *P. monodon* effector caspase (PmCaspase) by the WSSV protein anti-apoptosis protein 1 (AAP1 or WSSV449) has been shown in Sf9 cells [107]. AAP1 binds to, and is cleaved by, PmCaspase at two possible sites, with only one resulting in PmCaspase inhibition [107]. In a similar cellular system another effector caspase in *P. monodon*, PmCasp, can be bound by viral proteins WSSV134 and WSSV322, resulting in anti-apoptotic activity [108]. However, inhibition of PmCasp by AAP1 and WSSV449 does not occur, illustrating the diversity of effector caspases and their activity [108]. A different method of host caspase regulation has been revealed in *M. japonicus*. Here, the caspase gene *PjCaspase* was identified and shown to be upregulated in survivors of WSD [190]. Silencing of this gene resulted in inhibition of apoptosis, and the subsequent increase in viral copy number showed that induction of apoptosis in infected cells is beneficial to the host. Zuo *et al.* [145] have also shown that WSSV can regulate the expression of caspase. Viral proteins VP38 (WSV259) and VP41B (WSV242) were capable of binding the *PjCaspase* promoter, the first acting as a repressor and the latter acting as an activator. Interestingly, both of these WSSV proteins are envelope proteins: VP41B having a potential transmembrane domain and VP38 through association with envelope protein VP24 [191,192]. The capability of WSSV to both repress and promote caspase genes follows the observation that some viruses induce apoptosis to facilitate departure from the host cell.

Yet another route through which WSSV can induce apoptosis is through ubiquitination, a process by which attachment of ubiquitin to a protein can flag it for degradation in proteasomes. This mechanism plays an important role in regulation of apoptosis as many substrates of ubiquitination are regulatory proteins for apoptosis [193]. Ubiquitin is activated by an ubiquitin-activating enzyme (E1), subsequently transferred to a ubiquitin-conjugating enzyme (E2) and, through interaction with an E3-ligase, transferred to the target protein [194]. WSSV249 has been shown to be involved in ubiquitination by acting as an E3-ligase in cooperation with the conjugating enzyme PvUbc in *L. vannamei* [109]. This WSSV249 interaction is accomplished through its RING-H2 domain, a domain associated with E3-ligases [46,195]. RING domains are present in four WSSV proteins, namely WSSV199, WSSV222, WSSV249, and WSSV403 [109,196]. The RING-H2 domain of WSSV222 enables it to perform as an E3-ligase in the ubiquitination of tumor suppressor-like protein, thereby inhibiting apoptosis [197]. Whether any of the other RING domain containing proteins have apoptosis inhibiting function remains to be determined. Together with caspase inhibition, the success of ubiquitination of pro-apoptotic proteins will determine the fate of the host cell [187].

Wang *et al.* [110,198] have suggested a third virus–host interaction that could influence the apoptotic state of the host cell, involving the highly expressed WSSV protein ICP11. The crystal structure of dimers of ICP11 indicates that it could act as a DNA mimic [110]. The electrostatic surface of ICP11 shows patches of negatively charged amino acids that are arranged in two rows and at similar distances as dsDNA phosphate groups [110]. Furthermore, it was shown that ICP11 could interfere with the binding of host DNA to histones (H3) in HeLa cells [110]. This binding can lead to disruption of the host nucleosome assembly and even apoptosis [110]. An alternative crystal structure of the dimer of VP9 (ICP11) has been proposed that does not show such rows of negative charges [199]. Instead, VP9 shows structural folds that bear resemblance to E2, a transcription/replication factor of the human papillomavirus [111,200]. In *P. monodon* it has been shown that VP9 can interact with a receptor for activated protein kinase C1 (PmRACK1), using the yeast two-hybrid and GST pulldown assay, [111]. Mammalian RACK1 receptors are involved in a large variety of functions including cell signaling pathways, cell development, and the immune response, and can interact with a large number of viral proteins [111]. The interaction between these two proteins may be involved in intracellular VP9 functions, for example by transporting VP9 to the nucleus [111].

#### 3.4.5. Particle Assembly and Release

After replication of the viral genome and production of structural proteins, these components come together to be assembled into new virions. One of the most challenging aspects of viral particle assembly is packaging the WSSV 300 kb genome into the nucleocapsid. In eukaryotes, DNA is compacted in nucleosomes but this does not occur in viruses. In dsDNA viruses, DNA packaging is often accomplished through interactions between DNA and nucleocapsid proteins [201]. A small (6.7 kDa) viral protein, VP15, has been associated with WSSV DNA packaging. VP15 is a basic protein that shows homology to putative baculovirus DNA-binding proteins [202]. Studies have shown that VP15 can form homomultimers and is able to bind to (preferably supercoiled) DNA [202]. Application of Atomic Force Microscopy has shown that VP15 is able to condense DNA and that VP15-induced DNA condensates resemble packaged viral DNA [201]. In the packaging process VP15 can interact with host proteins. In *P. monodon* binding has been shown between VP15 and PmFKBP46, an immunophilins-like protein [112]. The role of PmFKBP46 in *P. monodon* is not known but human and yeast homologues are involved in histone deacetylation and act as a histone chaperone, respectively [112,203,204]. A more recent study uncovered an interaction between VP15 and calreticulin (CRT), which could also act as a histone chaperone [101,205]. DsRNA-mediated knockdown of CRT resulted in a significant decrease in viral DNA duplication and viral gene transcription, indicating that the interaction between CRT and VP15 is necessary for viral replication. It has also been suggested that the interaction between VP15 and CRT can play a role in the export of viral RNA from the nucleus, a role that CRT has been shown to fulfill for glucocorticoid receptors [101,206].

Morphogenesis of the WSSV envelope requires long chain fatty acids (LCFA). During the early stages of infection fatty acids in the host cell are depleted in order to generate the energy required for viral replication, but during the late stage of infection LCFAs are upregulated, thus replenishing the LCFA that have been used [207]. Applying an inhibitor of fatty acid synthase (FAS) that blocked the replenishment of LCFA was shown to result in impaired virion formation [207]. There is little information available on how assembled WSSV virions are subsequently released from the host cell.

## 4. Current Treatment Options for WSD

Despite the large body of work, our understanding of WSSV infection and pathogenesis are far from complete. Unresolved questions include those focused on mechanisms of endosomal escape, virion assembly, exit from the host cell, and the role of miRNA regulation. Nevertheless, because of the urgent need for disease mitigation (and prevention) avenues have been explored for potential therapy. Here we discuss some of the more promising results that include strategies spanning immune system activation, vaccinations, RNAi, and the application of herbal extracts.

Early studies investigated whether WSD could be prevented by enhancing immune competence of the host. This can be achieved by feeding with agents containing pathogen-associated molecular patterns (PAMP) that are known to activate the host innate immune system. It was found that feeding *P. japonicus* with peptidoglycans over a period of time increased the phagocytic activity of host granulocytes, resulting in a significant decrease in mortality upon WSSV exposure [208]. Similarly, injection of β-glucan prior to WSSV infection has been shown to result in activation of the prophenoloxidase system and subsequently a reduction of mortality (25%–50% as compared to 100% in controls). However, repeated dosages of β-glucan cause high mortality rates in the host, which is probably due to excess generation of reactive oxygen species [209].

Invertebrates do not possess an adaptive immune system and they thus rely on innate immune defenses. As a consequence, it is not possible to develop vaccines in the traditional way as is done in mammals and other vertebrates. However, recent developments have provided evidence that certain forms of pathogen-specific “immune priming” are possible in some invertebrates [210]. For example, short-term protection against WSSV can be achieved through exposing shrimp to inactivated viral particles [211]. Musthaq and Kwang reviewed the possibilities of such vaccinations for WSD and the types of treatments (vaccines) that can provide temporal protection against WSD [210]. These include inactivated virus, recombinant viral proteins, viral DNA, and double-stranded RNA, and often involve WSSV envelope proteins like VP28 because of their importance to the infection process. However, all of these treatments provide temporal protection that does not usually last beyond 14 days. New generation technologies are attempting to provide more efficient delivery systems for these treatments. For example, baculovirus and *Bacillus subtilis* spores, both modified to express VP28, could convey protection via oral vaccination [212,213]. Elucidation of the mechanisms underlying invertebrate immune priming could lead to further gains in vaccine efficacy and provide an optimized solution.

RNAi can potentially play a significant part in host–pathogen interactions. Hosts can express small RNA molecules that target and inhibit the expression of viral proteins. It has been shown that *M. japonicus* can generate small interfering RNA (siRNA) that targets *vp28* (*vp28-siRNA*) in response to infection by WSSV. Blocking siRNA synthesis resulted in increased viral copy numbers, indicating that RNAi had a protective effect for the host [214]. In other work *vp28-siRNA*s encapsulated with β-1,3-d-glucan and injected along with WSSV in *M. japonicus* [215] was shown to inhibit WSSV replication, illustrating a potential avenue for treatment development.

Another direction of research focused on combating WSD has been the utilization of plant extracts with potential pharmaceutical activity. Large screens with extracts from plants have been carried out in an attempt to identify chemicals with anti-WSSV properties and there have been a number of successes. Examples include extracts of *Cynodon dactylon* and *Ceriops tagal* [216,217] that have shown protective effects against WSSV in *P. monodon*. Extracts from the seaweed *Sagrassum weighti* have been shown to have a significant effect in reducing WSSV infection in both the Indian prawn (*P. indicus*) and freshwater crab (*Paratelphusa hydrodomous*) [218,219]. Oral administration of a plant extract from *Momordica charantia* has been shown to result in an 86% survivorship in *L. vannamei* infected with WSSV [219]. Furthermore, several diets based on extracts from *Agathi grandiflora* have also been shown to decrease mortality rates substantially in WSSV-infected *Fenneropenaeus indicus* [220]. The nature of the pharmaceutically active components of these extracts, and how they interact with host and virus, is unknown for the treatments described. Filling these knowledge gaps could result in a better understanding of WSSV infection and aid in optimizing efficacy in the use of these materials in preventing WSD.

## 5. Future Perspectives

In recent years WSD has received significant scientific attention, driven by the commercial impacts of this disease on shrimp. Global research efforts into understanding the biology of WSSV and infection process for WSD have contributed to the formulation of strategies for disease treatment and prevention. Molecular studies have identified a substantial number of WSSV envelope and cell surface proteins involved in the first stage of virus infection, established a large number of host transcription factors replication of WSSV, and established some of the mechanisms by which the virus maintains a favorable host cell environment including through arresting the cell cycle, changing metabolism, and preventing apoptosis. Major gaps, however, remain in our understanding of how the virus, upon entering the host cell via endosomes, subsequently delivers its genome to the host nucleus, the virion assembly process, and in fact the function of the majority of WSSV proteins.

Research on WSD infection has been complicated by the lack of well-annotated genomic resources for host species. There has been a reliance placed on sequence similarities with other (sequenced) species to provide required annotation. Sequence information for aquatic crustaceans, however, is extremely limited. Application of modern sequencing technologies now allows for relatively rapid generation of the required sequence information to support both *de novo* genome assemblies and for transcriptome analyses, and this work needs to be encouraged to provide the required molecular resources for commercially important species as a minimum. Current research on WSSV infection has focused overwhelmingly on susceptible shrimp species, which is not surprising given their economic importance, but opportunities could lie in studies on more resistant (and perhaps less popular) host organisms. Understanding how those organisms resist WSD could equally lead to effective disease treatments for shrimp.

Understanding the WSSV infection process completely may not be necessary in order to produce effective therapeutics. Current knowledge provides a plethora of potential pathways/genes/miRNAs that could serve as potential treatment targets and indeed there has been some success in applying targeted molecular approaches in the treatment of WSD. To date, however, these treatments applied in a laboratory context have not been successfully applied in the field. As an example, RNAi has shown great potential as therapeutic technology and indeed VP28-siRNA has been shown to be effective against WSSV. However, it requires delivery through injections, which is not viable for commercial application. The development of an effective delivery method for siRNA that can be used in shrimp farms would be a significant step forward in the prevention of WSD and would also have great potential for use in the treatment of other viruses that impact aquatic Crustacea. Other prospects include production of genetically modified shrimp for resistance to WSD, for example by enhancing anti-WSSV RNAi.

Another potential avenue for exploration in combating WSD relates to resistance that has developed in certain hosts through integration of viral DNA into the host genome, conveying resistance to the inserted virus (and sometimes also closely related pathogens). If such a mechanism of resistance could be transferred from resistant to susceptible shrimp species, this could result in the production of shrimp lines that are resistant to WSSV.

Bringing this review to a close, we would emphasize that while understanding the molecular basis of WSD is likely to lead to possible intervention strategies for combating WSD, the practical implementation of this knowledge, given the nature of the shrimp farming industry, will require the resulting treatments to be cheap, easy to use, and easily distributed. It should also be recognized that there are many other factors that need due consideration in our attempts to develop improved treatments for, and to combat, WSD. For example, it is the case that outbreaks of WSD in farming will inevitably depend on the health status of host organisms and the environments in which they live and this is not necessarily determined by individual pathogens alone but by a combination of local abiotic and biotic factors, pathogen assemblages, and pathogen loads in host tissues. Almost nothing is yet known in this regard for WSD. Indeed, WSSV may be endemic in aquaculture and may only occur under certain conditions. Identifying those conditions and the biological indicators associated with health status and disease outbreaks in shrimp aquaculture ponds could help in predicting and pre-empting WSD outbreaks, allowing for intervention strategies, and uncoupling the ability of WSSV to interact with its host. More effective and intelligent surveillance systems for preventing the spread of WSD outbreaks are also a major research need.
